# Training in health coaching skills for health professionals who work with people with progressive neurological conditions: A realist evaluation

**DOI:** 10.1111/hex.13071

**Published:** 2020-05-28

**Authors:** Freya Davies, Fiona Wood, Alison Bullock, Carolyn Wallace, Adrian Edwards

**Affiliations:** ^1^ Division of Population Medicine Cardiff University Cardiff UK; ^2^ School of Social Sciences Cardiff University Cardiff UK; ^3^ Faculty of Life Sciences and Education University of South Wales Pontypridd UK

**Keywords:** continuing professional development, health coaching, person‐centred care, realist evaluation, self‐management

## Abstract

**Background:**

Supporting people to self‐manage their long‐term conditions is a UK policy priority. Health coaching is one approach health professionals can use to provide such support. There has been little research done on how to train clinicians in health coaching or how to target training to settings where it may be most effective.

**Objective:**

To develop theories to describe how training health professionals in health coaching works, for whom and in what circumstances, with a focus on those working with people with progressive neurological conditions.

**Design:**

Realist evaluation using mixed methods (participant observation, pre‐ and post‐training questionnaires, and telephone interviews with participants and trainers). Realist data analysis used to develop and refine theories.

**Intervention:**

Two 1‐day face‐to‐face training sessions in health coaching with 11 weeks between first and second days.

**Setting and participants:**

Twenty health‐care professionals who work with people with neurological conditions in the UK, two training facilitators.

**Results:**

Four theories were developed using context‐mechanism‐outcome configurations to describe how training triggers critical reflection; builds knowledge, skills and confidence; how participants evaluate the relevance of the training; and their experiences of implementing the training. Some participants reported a major shift in practice, and others implemented the training in more limited ways.

**Discussion:**

Fully embracing the role of coach is difficult for health professionals working in positions and settings where their clinical expertise appears most highly valued.

**Conclusions:**

Training should address the practicality of using coaching approaches within existing roles, while organizations should consider their role in facilitating implementation.

## 1 INTRODUCTION

People with long‐term conditions make many daily decisions that affect their health.^1^ Supporting self‐management among people with long‐term conditions is high on the UK policy agenda.^2^, ^3^, ^4^ We know that people value health professionals who can provide tailored support specific to their unique circumstances, to help them effectively self‐manage their conditions.^5^, ^6^ However, this delegation of responsibility to individuals (often largely motivated by resource availability) can be burdensome.^7^, ^8^, ^9^ People living with progressive neurological conditions (PNCs) such as Parkinson's disease and multiple sclerosis experience complex interacting symptoms which may make it challenging for them to follow all the recommendations from their health‐care providers. For example, people with multiple sclerosis may find it difficult to engage in the suggested level of physical activity while simultaneously trying to manage heat sensitivity and fatigue.^10^, ^11^ Furthermore, the changing nature of PNCs often necessitates on‐going adaptations to daily life.^12^

Health coaching is one method of supporting self‐management and person‐centred care.^2^ Health coaches are trained in specific communication strategies and use behaviour change theories together with motivational techniques, to enlighten and empower the people they work with, aiming to foster people's intrinsic motivation.^13^, ^14^ Internationally, notably in the United States, there has been a focus on developing health coaches who work alongside other health‐care professionals.^15^ In the UK, there has been increasing interest in training a range of health professionals to integrate health coaching skills into their routine consultations.^16^, ^17^, ^18^ Health coaches are expected to hold an unconditional positive regard for those with whom they work and to believe in those people's expertise and capacity to change.^19^, ^20^ This approach contrasts significantly with traditional expert‐orientated models of care.

Health coaching is a complex intervention, and the implementation chain, towards improved health and well‐being, is long. The effectiveness of health coaching interventions is unclear, partly because of the variability in existing studies in mode of delivery, duration, intensity, characteristics of coaches and those being coached, which hinders conventional systematic reviews.^21^, ^22^, ^23^, ^24^ Research approaches that are designed to account for and understand this complexity are required to better understand health coaching interventions.

Training health professionals in coaching skills is the first essential stage of any health coaching intervention. There is currently a ‘dearth of research’, 162 pp., in relation to the training of health coaches.^25^ While there is general agreement that training should last at least 2 days, and involve opportunities to practise coaching and experience being coached,^26^, ^27^ previous evaluations have found that responses to training and subsequent implementation vary widely.^17^ To inform the design and tailoring of future programmes, and to assist commissioners in making decisions about what types of clinicians, working in which settings, the training might be most influential, we aimed to explore how staff working in the UK NHS with people with PNCs responded to 2 days of training in health coaching skills.

The two questions we aimed to address were as follows:

1. How does the training work? (What are the important resources provided by training and what reasoning does training trigger among participants?)
2. How does the training work differently when delivered to different clinicians working in different settings?

This evaluation took place as part of a wider PhD study exploring how training health professionals working with people with PNCs could improve self‐management support provision. Earlier stages of the PhD included a survey of 186 health professionals and a realist review of the literature relevant to training health professionals to support self‐management among people with PNCs.^28^, ^29^ This evaluation builds on the earlier findings (examining a range of possible self‐management support approaches) and explores their relevance to a specific health coaching intervention.

## 2 METHODS

We undertook a realist evaluation of a health coaching course delivered over 2 days (11 weeks apart) to 20 UK health professionals who work with people with neurological conditions.

Realist evaluation is a theory‐driven approach, sensitive to complexity, which focuses not on the average effectiveness of interventions, but on explaining the reasons why interventions work differently in different settings.^30^ As such, it was well suited to addressing the aims of the research, and likely to produce policy‐relevant findings. Realist researchers focus on building theories about causation. Interventions are understood to offer a certain set of resources, which are then introduced into a unique context. Contextual influences may include characteristics of individuals (e.g. level of enthusiasm), wider organizations (e.g. financial incentives) and other influences such as the history of the setting (similar interventions implemented in the past). Features of the context influence how individuals reason about the new intervention, and this reasoning process can then lead to or prevent outcomes of interest occurring. Theories which describe causation are presented using context‐mechanism‐outcome configurations which outline how the intervention mechanisms (a combination of the resources provided and the reasoning triggered), influenced by context, act to generate outcomes.^30^, ^31^

Realist researchers recognize that our understanding of causation is only ever partial, and work towards a better understanding through an iterative process of theory generation, testing and refinement.^30^ The evaluation reported in the current paper aimed to further test and refine the theories developed in our earlier realist review.^28^ The theories presented in our review are briefly outlined in Table 4 mapped against the theories developed in the evaluation stage reported here.

### 2.1 Recruitment

The training course was advertised through professional networks, emailing lists and at two national conferences. Expressions of interest were sought, including brief information about potential participants’ professional roles and patient groups. The relatively small number of applicants formed a reasonably mixed sample, so further purposive sampling was not pursued. There were 44 expressions of interest, 38 training spaces were offered and 21 of these offers were accepted. Six were not offered a space because the course had reached capacity. One participant was unable to complete training (their pre‐training data were excluded from analysis).

### 2.2 Ethics

Ethical approval was obtained from Cardiff University School of Medicine Research Ethics Committee (SMREC 17/66). Participants were made aware of the research project in the initial information they received about the training. Participation in the research was not a mandatory component of the training, but all participants agreed to be involved and provided written informed consent. Participants were given regular opportunities to decline to participate in parts of the evaluation in order to minimize any perceived coercion. Interview transcripts and field notes were anonymized for confidentiality. While the responses of other participants were discussed in broad terms during the realist interviews, anonymity was strictly preserved. Funding for the training was provided by an education grant from Novartis Pharmaceuticals. Novartis had no input into the training content, provision, evaluation or reporting.

### 2.3 The intervention

A more detailed description of the training intervention compiled using the Template for Intervention Description and Replication (TIDieR) checklist^32^ can be seen in Appendix 1. In brief, training was provided during two 1‐day sessions in early 2018 by an external training provider with extensive experience of working with NHS staff. Usually, the second day is delivered after a 2‐ to 4‐week gap to allow participants time to try the techniques in their clinical practice, but due to severe adverse weather the second training day was postponed resulting in an 11‐week gap. Two trainers with clinical backgrounds facilitated the highly interactive course which they conceived and developed and have been running for several years. The approach combines executive coaching skills, behaviour change skills and clinical communication skills. Training included short presentations, coaching demonstrations, discussions with other participants and working in pairs to practise coaching and being coached using a variety of techniques. The training covered a range of specific coaching techniques as well as introducing other behaviour change strategies and topics relating to self‐management support (see Table 1).

**Table 1:** Training programme content^33^

Core training topics	Specific techniques
The coaching mindset and approach	TGROW (topic, goal, reality, options, will/way forward) model^34^
Directive and non‐directive approaches	Diamond model
Goal setting	ABC (antecedents, behaviour, consequences) model^34^
Using coaching in a clinical setting	Solution‐focused coaching^35^
Patient activation^36^	Brief motivational interviewing^37^
Using challenge	Managing interferences using coaching
Transactional analysis^38^	
Stages of change^39^	

### 2.4 Data sources

Realist evaluations typically use a mixed‐methods approach, recognizing that different types of data can provide insights into different elements of context, mechanisms and outcomes.^40^ Table 2 summarizes the data sources and the rationale for each chosen approach.

**Table 2:** Evaluation data collected and rationale

Time point		Data collected	Rationale
Pre‐training	January 2018 (immediately before training started)	20 questionnaires (100% response rate)	Demographic data to improve understanding of context Provide baseline data for comparison
During training	January 2018 and April 2018	Observations of 2 full days of training (20 participants, 2 trainers)	Provide researcher with best possible understanding of the intervention Researcher personally experiences training mechanisms Improve the quality of the telephone interviews due to researcher familiarity with training content and participants
Post‐training	April 2018	20 questionnaires (100% response rate)	Identify key training outcomes Immediate post‐training data for comparison to pre‐training ratings
	From 10 d to 7 wk post‐training (17/19 within 4 wk)	19 participant interviews	Improve understanding of individual and workplace context influencing response to training Explore training mechanisms Discuss theories in development for refinement
Follow‐up post‐training	12‐24 wk post‐training	13 questionnaires (65% response rate)	Identify whether immediate post‐training outcomes were maintained and whether the impact of training appeared to increase following further experience of implementation
	14‐24 wk post‐training	11 follow‐up participant interviews	Discuss experiences of implementation Discuss theories developed from earlier data to aid theory refinement
	September‐October 2018	2 trainer interviews	Discuss theories in development for refinement Provide insights from experiences of training outside the course evaluated to assess transferability of findings

### 2.5 Observations

FD, a practising clinician (general practitioner) and researcher, acted as a participant observer. Field notes were taken during both days and written up in detail shortly after the training while also referring to the administrator's notes (DE). These included observations of participants’ reactions to each activity and the responses offered during group discussions. Personal insights and interpretations of the researcher were separately recorded.^41^

### 2.6 Interviews

FD conducted all interviews and was already known to participants from the training. A realist approach to interviewing was used in which the theories under development were the main focus of the discussion.^30^, ^42^ Theories developed from our earlier review^28^ were used to develop a topic guide. A teacher‐learner style was used, especially in the later interviews when descriptions of the researcher's theories in development were ‘taught’ to participants, with the researcher seeking to ‘learn’ how these fitted with individuals’ experiences.^30^, ^42^ The focus of the interviews therefore changed depending on the stage of theory development that had been reached, and the particular theories to which individual participants were expected to be able to contribute data. Both trainers were also interviewed. The interviews were audio‐recorded and transcribed verbatim.

### 2.7 Questionnaires

Bespoke questionnaires were designed and delivered at three time points: immediately pre‐training, immediately post‐training (paper‐based) and 3 months post‐training (online). The questionnaires used seven descriptors of key health coaching skills used by the training company in their own evaluations and asked participants to rate their current understanding, confidence in using and extent of use of each technique on a 5‐point Likert‐like scale (see Box 1). It also asked fixed‐response questions about perceived importance, usefulness, ease of application and motivation to use health coaching techniques. Additional free‐text items allowed participants to elaborate further. The initial questionnaire included supplementary information on participant demographics. The participant's study ID was included in each questionnaire to allow changes in individuals’ responses to be tracked over time.

Box 1 **Health coaching skills assessed in the questionnaires^43^**

**Table:** 

**Focusing on patient's goals**—understanding what the patient really wants to achieve and developing commitment to those goals more than the focus on your own clinical objectives **Demonstrating empathy**—aiming to understand the patient's context by putting yourself ‘in their shoes’ **Raising awareness**—asking questions that encourage your patients to develop new insights that support self‐management **Encouraging responsibility**—supporting patients to take responsibility for their own management rather than relying on your advice **Supportive challenge**—challenging the ideas and perspectives of your patients in a supportive manner **Awareness of self**—monitoring your own thoughts and feelings during consultations, being aware of judgements and habits **Patient resourcefulness**—communicating in a way that conveys confidence, respect for and belief in the patient's ability to be resourceful

### 2.8 Data analysis

A triangulation approach was used with the qualitative and quantitative data being analysed concurrently by FD, (who has expertise and training in qualitative and realist methods).^44^, ^45^ The quantitative data analysis focused on generating descriptive statistics and identifying changes in individual participants’ questionnaire responses over time.^40^

All qualitative data were imported into NVivo 11. An initial set of codes was generated using the theories from our review^28^ and supplemented by additional codes developed from a reflective journal kept by FD during the interview stage. Further codes were inductively developed during the initial coding of the qualitative data from the questionnaires and the first five interviews.^46^ After initial ‘first pass’ coding of all questionnaire and initial interview data, the coding framework was further refined, with some codes merged. An audit trail of all decisions was maintained. ‘If‐Then’ statements were generated after exploring the coded data, initially at a lower level, close to the data, before being grouped together into related topic areas.^47^ Four topic areas were developed which were used to generate higher‐level theories, described using context‐mechanism‐outcome configurations. The qualitative data were reviewed alongside the quantitative data at this stage, with a focus on exploring the reported outcomes of the training.

### 2.9 Enhancing rigour

This article was prepared with reference to the RAMESES II publication guidelines for realist evaluation.^48^ The research was theory‐driven, based on the findings of our earlier literature review.^28^ Triangulation of data sources and data collection methods provided a more comprehensive understanding of how the training worked, and allowed convergence of the results to be identified.^49^, ^50^ The learner‐teacher approach to interviewing meant that participants were able to refute and refine the researcher's theories.^30^, ^42^ Attention was also paid to the role of the researcher as a health professional, and the way in which this may have influenced the data collected and the interpretations made. For example, being observed by a colleague may have influenced participants’ behaviour towards supporting the intervention. While being a clinical researcher facilitated rapport building due to shared understandings, it also increased the risk of making assumptions or missing the obvious. Regular meetings between all authors were held throughout the study and emerging findings discussed. Data extracts are presented with the results below to allow the reader to judge the inferences made.

## 3 RESULTS

Twenty participants completed the two training days. The professional backgrounds of the participants are shown in Table 3. Complete data (completed interview and questionnaire) were available from 95% of participants at the immediate post‐training stage and from 40% of participants at the follow‐up stage (with a further 40% providing partial data). Nineteen participants were female. Sixty percent had worked in neurology for more than 10 years. Nine participants attended the training alone and 11 with someone else from their organization. Two participants worked with people with non‐progressive neurological conditions. They were invited to attend as several other members of their team were also attending, and it was felt that training a large team together could help to develop the theory about the importance of team support. Half of the participants worked with people with a single neurological condition, while half worked with people with a range of different neurological conditions. One participant was employed by a third‐sector organization, and the remainder all had NHS roles.

**Table 3:** Professional background and experience of participants

Background	Number of participants (% of total participants)	Time working in neurology setting (range)
Nursing	5 (25%)	Between 7‐9 and 10 y or more
Physiotherapy	5 (25%)	Between less than 1 and 10 y or more
Occupational therapy (currently working in therapist role)	5 (25%)	Between less than 1 and 10 y or more
Occupational therapist (currently working as clinical specialist)	4 (20%)	Between 1‐3 and 10 y or more
Speech and language therapy	1 (5%)	10 y or more

We produced four refined theories describing how the training works, for whom and in what circumstances which are presented below. The content of each theory is first briefly described, and the context‐mechanism‐outcome configuration generated is then presented, followed by some of the evidence used to develop this theory. Box 2 explains the labels used within the theory statements. Table 4 shows how the theories build upon and relate to the earlier work.

**Table 4:** Theory development

Summary of theory from our earlier review^28^	How this advanced in our evaluation theory
Evidence—*may be needed to convince professionals to change their approach*	Critical reflection Evidence provision is one trigger of critical reflection Relevance to setting Evidence can legitimise taking a new approach
Knowledge, skills, confidence and self‐efficacy—*are developed during training to enable provision of effective support*	Knowledge, skills and confidence Opportunities to practise specific techniques in a safe space increase confidence
Reflection—*on personal effectiveness (triggered by training) can motivate practice change*	Critical reflection Coaching and being coached triggers reflection on usual style
Empathy—*is developed during training and results in changed expectations of patients*	Critical reflection Reflection on consultation style develops empathy Experiences of implementation Trying out the new skills changes interactions, and these different conversations can trigger increased empathy among professionals
Team and organizational support—*influence how professionals conceptualize their role in relation to self‐management*	Relevance to setting Organizational factors can hamper integration of new skills Experiences of implementation When a coaching approach ‘fits’ with the existing team ethos, it is easier to implement
Redefining professional role—*training works by making professionals see their role differently*	Relevance to setting Re‐evaluating what patients need can lead to a change in view of own role
Picking the right patient—*professionals support self‐management selectively, based on their own assessment of the relevance to each patient*	Knowledge, skills and confidence Perceived levels of knowledge, skills and confidence depend on the complexity of the patient's needs Relevance to setting Perceived patient‐level barriers influence how relevant professionals believe the skills to be Experiences of implementation Trying out the new skills with patients informs views on when they might or might not work in future

Box 2 **Definitions of context, mechanism and outcome labels^31^**

**Table:** 

Context (C)—the situation into which the intervention is introduced Mechanism resource (Mresource)—the resources introduced into the context by an intervention Mechanism reasoning (Mreason)—the subsequent change in reasoning that occurs Outcome (O)—generated by the introduction of intervention resources, into a context which triggers a reasoning process

### 3.1 Theory 1: critical reflection

This theory describes how training led participants to critically reflect on their current approach and their need to change their practice.

*Training activities, along with interactions with colleagues and trainers (Mresource) help participants to develop greater self‐awareness, and improved understanding of how others work and the impact of their own consulting style, and to recognise the benefits of a health coaching approach (Mreason). These training experiences lead participants to develop a new view on their own role, and the skillset they require (O). The creation of a safe training space facilitates this reflection (Mresource). Participants who attend training because it meets a pre‐identified learning need are more receptive to the training (C). Those more concerned with issues outside their own control (patient and organisational factors) appear less critically reflective about their own performance (C)*.

The training activities, including opportunities to watch coaching demonstrations, participate in role play and have discussions with colleagues all acted as triggers for participants to reflect on their current approach. Participants already recognized that their current consulting styles were not always successful, and training prompted participants to identify what it was that was less effective. For some participants, this triggered self‐reflection and an interest in changing the role they adopted during consultations (which they recognized would require developing new techniques).

P2: I’m very eager to please and fix things, so learning not to do that, I can't say I've stopped doing that, but realising there’s more, there’s more, there must be more to my interventions than doing that. (initial interview)

Participants with this type of response had often already spent time before the training reflecting on their training needs and appeared ‘primed and ready’ for training. The trainers agreed that those attending with an identified skills deficit were usually most receptive.

Some participants, while recognizing their own deficits, remained focused on the wider barriers to successfully supporting self‐management. These included perceived barriers at the patient level and competing organizational priorities.

P1: Once it started to come to light, in the first day, you identified what your style was and how you could change it, I think the time constraint is probably the biggest challenge really. Because, at the end of it all you have a proforma that has to be ticked for auditing processes, a letter has got to be generated, and you’ve got people sitting outside. (initial interview)

The data suggested there was an interaction between different elements of context (individual and organizational factors), but it was not possible to identify the relative influence of the different elements with the data available.

### 3.2 Theory 2: Knowledge, skills and confidence

This theory describes how the training process builds participants’ knowledge, skills and confidence.

*Providing a safe and authentic environment in which to learn and practise new skills (Mresource), and experience success (Mreason), allows participants to become more confident in their understanding of what doing health coaching means for them and in their own ability to implement health coaching (O). When training is experienced negatively (Mreason), because it highlights a skills deficit, fails to create a feeling of safety or appears impossible to integrate into routine care, participants lack confidence in their own ability to implement health coaching (O). Low pre‐existing confidence levels, or existing views on patient, team and organisational expectations may make it more difficult to develop confidence in the new approach (C)*.

The questionnaire data showed 90%‐95% of participants’ self‐reported understanding of health coaching techniques and confidence in using them improved immediately post‐training (see Table 5). However, not all participants maintained these improvements when surveyed again after 3 months. The qualitative data provided a more nuanced understanding of how knowledge and confidence were built. While all participants talked about having more techniques to draw on following the training, their confidence and motivation to implement these appeared more mixed.

**Table 5:** Quantitative results summary

	Pre‐training scores (range among 20 participants)	Immediate post‐training scores (range among 20 participants)	% of participants with improved immediate post‐training scores (20 participants)	Three‐month follow‐up scores (range among 13 participants)	% of participants with decreased scores from immediately post‐training to follow‐up (13 participants)
Understanding of health coaching techniques (mean scores across 7 techniques) (Likert scale 1‐5, 1 = do not understand at all, 5 = understand completely)	2.14‐4.14	3.29‐5.00	90% (increases in mean score on Likert scale up to 0.5 = 20% , 0.5‐1 = 35% , 1‐1.5 = 20% , 1.5‐2 = 10% , 2‐2.5 = 5% )	2.86‐4.85	76% (mean score on Likert scale decreased by up to 0.5 in 54% and between 0.5 and 1 in 23%)
Confidence in using health coaching techniques (mean scores across 7 techniques) (Likert scale 1‐5, 1 = not at all confident, 5 = extremely confident)	2.00‐3.57	3.14‐4.43	95% (increases in mean score on Likert scale up to 0.5 = 15% , 0.5‐1 = 45% , 1‐1.5 = 30% , 1.5‐2 = 5% )	2.71‐5.00	46% (mean score on Likert scale decreased by up to 0.5 in 23% and between 0.5 and 1 in 23%)
Perceived usefulness of health coaching (Likert scale 1‐5, 1 = not useful at all, 5 = extremely useful)	4.00‐5.00	4.00‐5.00	10% (increased by 1 on Likert scale)	3.00‐5.00	39% (decreased by 1 on Likert scale)
Perceived ease of use of health coaching (Likert scale 1‐5, 1 = very difficult, 5 = very easy)	2.00‐5.00	2.00‐5.00	21% (10.5% increased by 1 on Likert scale, 10.5% increased by 2)	2.00‐4.00	39% (15% decreased by 1 on Likert scale, 23% decreased by 2)
Motivation to use health coaching techniques in routine appointments (Likert scale 1‐5, 1 = not at all motivated, 5 = extremely motivated)	3.00‐5.00	4.00‐5.00	25% (15% increased by 1 on Likert scale, 10% increased by 2)	3.00‐5.00	69% (54% decreased by 1 on Likert scale, 15% decreased by 2)

The perceived authenticity of the training experience acted to facilitate or inhibit skills development. Some participants found features that increased the authenticity of the training also increased their own belief in the health coaching approach (e.g. the clinical experience of the trainers, practising coaching using personal examples from their own lives).

P17: They were obviously clinicians as well so it felt like they understood the issues that we might come across. (initial interview)

Experiencing the benefits of being coached, even by a ‘novice’, helped participants to feel more confident in their own practice.

P9: As a coachee I came away feeling like I had got something from being coached and so it was really heartening to feel that actually even if you don’t have all the skills, or you don’t feel totally that you are practised with them, just implementing the principles can lead to change (initial interview)

For some, discussing real‐life issues enhanced authenticity but also threatened the feeling of safety of the training. This led to a few participants feeling uncomfortable with discussing personal issues or to them choosing more superficial topics, which limited the impact of the experience of being coached.

P15: Because I felt they [topics participant chose to discuss] were fairly superficial I didn’t feel that – and because they’d been brought up in a slightly artificial situation that I didn’t necessarily feel that committed at the end of it, but then thinking about… the topics of one of the people I talked to used I felt she was very committed to what she was going to do afterwards (initial interview)

Participants who were less experienced in their current roles reported finding it easier to prioritize the ‘medical’ aspects of the consultation. Some described that reverting to information provision was easier than trying the coaching approach.. Some more experienced practitioners who were confident in their roles appeared to find it easier to accept a coaching role, which emphasized their medical expertise less.

P7: I think if you’re not so confident, or you’re, then you feel that you’ve got to solve it [the patient’s problem], or sort it and actually you haven’t (follow‐up interview)

The development of confidence was closely linked to perceptions about how the approach could be used, and challenges related particularly to the PNC setting.

### 3.3 Theory 3: Relevance to setting

This theory describes how the participants evaluate the relevance of the training to their own clinical setting.

*During training participants weigh up how useful they believe a health coaching approach is and how easy it would be to adopt, and this results in motivation (or lack of motivation) to apply the training in practice (O). Modelling of coaching by the trainers, provision of evidence for the approach and experiencing coaching (Mresource) can all highlight the usefulness of health coaching (Mreason). Training is perceived to be more useful when the approach also fits with pre‐existing ideas about professional role and meets a recognised learning need (C). Participants also evaluate how easy it will be to apply health coaching, influenced by interactions during training, (Mresource) and perceived fit with existing working practices and caseload demands (C)*.

While the importance of evidence was highlighted in the earlier review,^28^ the participants rarely brought up the evidence base for health coaching. When exploring the influence of evidence, ‘evidence‐based practice’ was recognized as a gold standard, but personal evidence was often cited as more influential.

P6: Obviously it’s a good thing if something is evidence‐based, if I find that I can actually apply it and get positive results with my patients then to me that is the most important thing (follow‐up interview)

Research evidence took on increased importance if participants needed to justify new practices to colleagues. As well as gathering personal evidence for the effectiveness, participants also made judgements about perceived usefulness for their patient group and fit within their existing routines. For those who worked in roles where medical or technical tasks were often the focus of the consultation, it was harder to be sure how to integrate coaching and ‘role conflict’ could occur when the coaching approach was not seen to fit with other tasks they were expected to complete. ^51^

P10: If someone is presenting with pain or spasticity and swallowing issues that they don’t know what needs to be done, or what medication needs to be prescribed … but when it’s more about talking to them about physiotherapy and exercise and lifestyle changes, that I think is where the coaching will come in a little bit more (initial interview)

Therapists who were familiar with goal setting and challenging their patients, and who frequently discussed lifestyle changes with their patients, appeared to see most easily how coaching aligned with their existing roles.

Some participants expressed concerns that for their caseload of people with PNCs, expecting people to take an active role in self‐management could be unrealistic. They emphasized that a level of acceptance and insight was required for these sorts of approaches to work.

P11: Some people don’t want to have MS they don’t like you for telling them they’ve got it and they want you to take it away and moving them forward from that is really tricky (initial interview)

Some raised concerns that the training had inadequately prepared them to deal with more challenging scenarios, such as using coaching with people with mental health problems or cognitive impairment. The trainers’ use of coaching techniques when queries were raised appeared to leave some participants with unanswered questions. Other trainees were already confident in working with these patient groups, and this seemed to help them feel confident to try coaching.

Participants suggested it was helpful for teams working together to have a shared understanding of coaching, and it could be difficult to use a coaching style when working directly with colleagues who used an alternative more traditional approach.

P14: what I found difficult was being in a clinic with a physio, because I do joint clinics with a physio and they didn’t, others hadn’t always been on the training so that was quite hard (follow‐up interview)

Many participants were expected to provide in‐service training to colleagues when they completed the training course, and this had raised awareness and encouraged participants to revisit the learning resources provided. Some reported using existing resources within their teams (e.g. access to clinical psychologist support) to continue to build their skills. It was also helpful if supporting self‐management was identified as a local priority. As shown in the quote from Participant 1 above (Theory 1), the need to prioritize the completion of mandatory assessment forms which did not easily accommodate a coaching approach was cited as a barrier to implementation.

### 3.4 Theory 4: Experiences of implementation

This theory describes participants’ experiences of trying to implement the training in routine practice.

*Experiencing success when trying out health coaching in practice, leads trained participants to re‐evaluate their previous practice (Mreason) and increases how useful they believe the health coaching approach to be and their own confidence in their developing skills (O). In order to implement training, participants must first be adequately motivated and confident, and identify appropriate low risk opportunities to practice (C). Participants also need to become convinced that health coaching can fit within their role (O), which may happen more in situations where they perceive coaching to be a flexible intervention (Mreason), and they have adequate team support (C)*.

For participants to experience success, they needed to try out using a coaching approach. The interview data indicated that while some motivated individuals made changes soon after training, others found disrupting their usual routines more challenging. Somewhat unexpectedly, even those who were low in confidence in their own skills often chose to try out coaching for the first time with patients who they were most struggling to support effectively. These scenarios seemed to offer a low‐risk way to trial the new approach as participants had already accepted that their current way of working was ineffective. It was in these scenarios that many most valued the coaching techniques.

P6: for patients that I see that tend to throw up barriers to everything they want to do, I feel like I have a tool that I can work with them and apply it, and that’s been really helpful, it’s nice to know that I’ve got that if I need it (follow‐up interview)

When participants tried a coaching approach and observed how people responded, this could trigger significant reflection about the deficits in their previous approach. Those participants who experienced this type of transformative learning^52^ described a move towards seeing patient engagement as a more co‐constructed process,^53^ and they started to understand the influence of their own behaviour.

P14: we do get the same patients sometimes coming through and I think sometimes we think ‘it’s them’, and I do, that’s my kind of shifting thought now is – is it because it’s them? or are we actually giving them any responsibility over their health? (initial interview)

The shift in awareness about the influence of their own approach on subsequent engagement appeared important in motivating continued use of health coaching skills and techniques following training.

While the follow‐up questionnaire data were incomplete (65% response rate), they indicated that the benefits of the training were not maintained for all (see Table 5). Decreases in the ratings for confidence (46%), perceived usefulness (39%), perceived ease of use (39%) and motivation to apply the techniques (69%) were reported when compared with their immediate post‐training rating (see Table 5). This may reflect how challenging some participants found it to apply coaching techniques and may also have been influenced by a lack of on‐going support post‐training.

Most participants were highly autonomous practitioners who had long appointment slots and could control how their work was organized to a certain extent. Even in these circumstances, which the earlier literature review^28^ had suggested would be favourable, not all participants appeared to integrate coaching techniques to a significant extent. While autonomy meant participants could create opportunities to try out coaching, it also often meant that they lacked the naturally occurring peer support that could be present in teams who work more closely. One large team attended the training together, but because all members worked independently there was little integration of coaching into their ‘team’ approach.

P2: we’ve all got our very separate caseload… generally we don’t have any overlap… I talk more to the physios and the OTs [occupational therapists] and the speech therapists, rather than my other colleagues within the team. I guess because they’re parallel, we work parallel (follow‐up interview)

### 3.5 An overall programme theory

**Figure 1:**
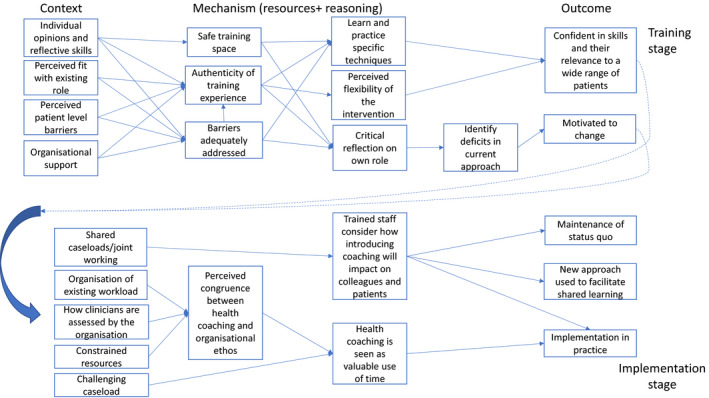
Overall programme theory

Figure 1 acts to summarize the findings of the evaluation, identifying key contexts, mechanisms and outcomes at both the training and implementation stages.

## 4 DISCUSSION

The evaluation has helped to refine four theories describing how health professionals respond to training in health coaching. These describe the important process of critical reflection on current practice and how training facilitates this; the other factors that influence the judgements that participants make about the value of the training (including work context and patient group); how participants build confidence in the new approach; and how this confidence is built, maintained or lost during attempts to implement the training in practice. The opportunities to practise coaching and to be coached were cited by all groups as key to both developing an understanding of the approach and building confidence.

The realist lens applied during this evaluation highlighted the importance of recognizing that training happens in a context and participants are continually making judgements about the fit between their own personal context and the approaches advocated by the training. Significant tensions were identified as practitioners attempted to move towards a more person‐centred approach which emphasizes the expertise held by the individual, while also trying to understand what this meant for how they used their own expertise. Other health coaching intervention studies found that some professionals reported already using a biopsychosocial approach and felt coaching aligned well with their role, while others who conceived their roles to be about providing professional advice and who wished to do what they felt was best for their patients found a coaching approach more challenging to integrate.^16^, ^18^, ^54^

The way in which services are organized and audited provides clear messages about the value of professional expertise. The tendency to prioritize ‘medical or technical tasks’ seen in this study may reflect the lack of routine measurement of person‐centred care, with work that is audited seen as the highest priority.^55^ Prioritization of person‐centred approaches by organizations influences how individuals prioritize these activities.^56^ Organizations have an important role in promoting person‐centred approaches as ways of completing routine work, rather than extra activities required in addition to other clinical tasks.^57^ When coaching is seen as a way to manage demand and to work more successfully when people appear ‘stuck’, then individuals may be more receptive to integrating the approach.^17^ Perceived patient‐level barriers to promoting a self‐management approach identified here and elsewhere included co‐existing physical and mental health problems^16^, ^58^ and wider social context (such as a lack of social or economic resources).^16^, ^58^, ^59^

This realist evaluation used a theory‐driven approach to test and refine a set of explanatory theories developed from the wider self‐management support literature in the setting of a health coaching training intervention.^28^ Key training mechanisms and the contexts in which they are facilitated or inhibited have been described. While multiple data sources were used, increasing the trustworthiness of the findings,^49^ evaluating the training across a range of different settings may have yielded different theories. Loss of respondents at follow‐up made understanding implementation patterns more difficult. This might reflect a lack of interest in the research project, or in the training itself, or relate to competing demands for time. There is a risk that because only those participants most enthused by the training may have continued to engage with the evaluation, those for whom the training was less impactful, or who experienced significant barriers to implementation may have been under‐represented. The evaluation of outcomes was limited to self‐reported data, and social desirability bias may have led to a tendency to positively evaluate the training and its impact on their clinical practice.^60^ Response shift bias may have led to an underestimation of the effectiveness of the training as participants may have rated their knowledge and confidence as higher pre‐training based on their incomplete understanding of the training content.^61^ We therefore recognize that the theories presented remain partial, and in line with the realist approach, new evidence could lead to further theory development. Further objective assessment of professional behaviour change and of subsequent patient‐level outcomes is needed to further develop the theories proposed. Research to clearly define the desired outcomes of integrating coaching into routine care from the perspectives of a range of stakeholders could help inform future evaluations.

## 5 CONCLUSIONS AND RECOMMENDATIONS

Specific training in using health coaching techniques to make consultations more person‐centred was highly valued by participants. However, for some, training alone did not create sufficient confidence in the new techniques or in their relevance to the participants’ roles.

During the training stage, participants need to become convinced that the training is relevant to their setting. Providing clearer guidance on how a coaching approach can be incorporated into existing roles and routines may be important.^17^, ^54^ While modelling a coaching approach during the training can be valuable (including encouraging participants to generate their own solutions) constraints must also be adequately explored to avoid generating frustration.^62^ This could involve discussing the potential patient and organizational‐level barriers identified by participants in more depth. Organizations should be aware that existing working patterns, team configurations and audited work may influence how relevant participants perceive the training to be and modifications could help maximize implementation. Organizations should also consider how they can create opportunities for peer support and on‐going reflection on training to build participants’ confidence and facilitate positive experiences of implementation of the new approach in routine practice.

## CONFLICT OF INTEREST

The authors have no conflict of interest to declare.

## AUTHOR CONTRIBUTIONS

FD originally conceived the study, collected and analysed the data and prepared the first draft of the manuscript. AB, AE, CW and FW provided advice on the study design and data analysis and revised the manuscript.

## Data Availability

Research data are not shared.

## APPENDIX 1 Completed TIDieR checklist for intervention reporting^32^

**Table:**

Brief name		
1	Provide the name or a phrase that describes the intervention	Health coaching skills development programme
Why		
2	Describe any rationale, theory or goal of the elements essential to the intervention	Trainers try to model a coaching approach during the training by encouraging participants to identify their own challenges and generate their own solutions Development of a coaching mindset—exploring what coaching is, how it differs to other types of relationship Opportunity to experience being coached and being a coach Development of particular coaching skills and techniques Opportunities to discuss how coaching skills could be used in practice
What		
3	Materials: Describe any physical or informational materials used in the intervention, including those provided to participants or used in intervention delivery or in training of intervention providers. Provide information on where the materials can be accessed (eg online appendix, URL)	Topics and techniques covered are outlined in Table 1. All trainees were provided with a 123‐page resource guide (which included space for notes). The booklet included all of the slides presented by the trainers during the two workshops (and some extra slides that were not discussed during the training days) Participants were encouraged to write in the resource guides
4	Procedures: Describe each of the procedures, activities and/or processes used in the intervention, including any enabling or support activities	Personal reflection exercises Discussions in pairs, small groups and as a whole Group work with flip charts Short presentations given by trainers Live demonstrations provided by trainers Practise sessions with colleagues Very limited individual feedback on performance Activities often physical—involving walking around the room as a group to discuss different flip charts pinned on the walls
Who provided		
5	For each category of intervention provider (eg psychologist, nursing assistant), describe their expertise, background and any specific training given	The training was provided by two highly experienced facilitators (both with clinical backgrounds)
How		
6	Describe the modes of delivery (eg face‐to‐face or by some other mechanism, such as Internet or telephone) of the intervention and whether it was provided individually or in a group	Face‐to‐face training course Supplemented by the availability of an online closed group forum which provided reference material and discussion boards
Where		
7	Describe the type(s) of location(s) where the intervention occurred, including any necessary infrastructure or relevant features	Delivered in a meeting room of a hotel, seating in a U‐shaped layout. Slides displayed on a screen and flip chart used by facilitator
When and How much		
8	Describe the number of times the intervention was delivered and over what period of time including the number of sessions, their schedule and their duration, intensity or dose	Delivered over 2 whole days just over 11 wk apart (training commenced at 9.30 am and finished just after 5 pmwith 50 min lunch break, 2 short coffee breaks of 10‐15 min) just over 6 h Day 2 had same start time, finished at 5 pm, lunch break 40‐45 min, tea breaks shorter—10 min am, 5 min pm)
Tailoring		
9	If the intervention was planned to be personalized, titrated or adapted, then describe what, why, when and how	Intervention encouraged participant interaction. Group discussion sessions were shaped by the issues raised by the participants and felt to be most relevant to them
Modifications		
10	If the intervention was modified during the course of the study, describe the changes (what, why, when and how)	The training is usually delivered with a 4‐wk gap between the two sessions. Due to adverse weather, the second training day was postponed resulting in a gap of just over 11 wk between the first and second training days. Due to the long interval between the two training days, the trainers arranged to host a one‐hour refresher webinar 10 d before the second training day which was attended by 6 participants. This provided an opportunity for attendees to reflect on their experiences with trying to implement the training and to revise content from the first training day. Other participants had the opportunity to watch the webinar recording online
How well		
11	Planned: If intervention adherence or fidelity was assessed, describe how and by whom, and if any strategies were used to maintain or improve fidelity, describe them	No planned fidelity assessment
12	Actual: If intervention adherence or fidelity was assessed, describe the extent to which the intervention was delivered as planned	Majority of slides were discussed in the training day. Trainers choose to use resources flexibly according to needs and responses of group
